# Supplementary material for: Diffusion Limitations and Translocation Barriers in Atomically Thin Biomimetic Pores
Source: Entropy (Basel). 2020 Nov 20;22(11):1326. doi: 10.3390/e22111326 (PMC7712548; doi:10.3390/e22111326)
Supplement: Supplementary file 1 [file entropy-22-01326-s001.pdf]

entropy

MDPI

Article

Diffusion limitations and translocation barriers in atomically thin biomimetic pores—Supplementary Material

Subin Sahu[1,2,3] and Michael Zwolak[1,*]

1 Biophysical and Biomedical Measurement Group, Microsystems and Nanotechnology Division, Physical Measurement Laboratory, National Institute of Standards and Technology, Gaithersburg, MD 20899, USA
2 Institute for Research in Electronics and Applied Physics and Maryland NanoCenter, University of Maryland, College Park, MD 20742, USA
3 Department of Chemical and Biological Engineering, University of Colorado Boulder, Boulder, CO 80309, USA
* Correspondence: mpz@nist.gov

Here, we give additional results, including figures that show the behavior across the full range of parameter space (e.g., versus voltage and strain) that we study.

IV characteristics: We now look at the consequence of diffusion limitations and translocation barriers in the IV characteristics of the pore. In Figure S1, we plot the IV characteristics along with fits of the form $I = a\,V^b$, where a and b are positive constants. For $q_O = -0.24\,e$, the best fit gives an Ohmic to slightly superlinear relation. This is expected when the current is mostly limited by large translocation barriers. The fit is poor, however, particularly for the 0 % strain case. This is a consequence of the highly competitive nature of transport in this case, where dehydration and electrostatic interactions are giving a fairly complicated free energy landscape, on top of which voltage eventually—at $V_{ext} = 1$ V—washes out the free energy features and the current becomes much larger than an Ohmic relation would predict.

For the $q_O = -0.54\,e$ pore, we see three different regimes of IV characteristics depending on the strain. For small strain, the current is super-linear, i.e., $b > 1$; for large strain, the current is sub-linear, i.e. $b < 1$; and, at some intermediate strain it is nearly linear $b \approx 1$. If the current is fully diffusion-limited, then we should get $b = 0$. However, the current we observe is not fully diffusion-limited, i.e., it does increase with voltage, but sub-ohmically (see Figure S1). Nonetheless, the weak dependence of current on the voltage at larger strain, with the channel tending to empty out as voltage increases, implies that the current has diffusion limitations. This kind of saturation, where the channel empties out as voltage increases, is similar to that in the MaxiK channel [1].

The diffusion limitations and translocation barriers have opposite effects in the IV characteristics. The diffusion limitations will make the IV characteristics sublinear (less dependent on voltage). Translocation barriers, on the other hand, will make the current superlinear because the applied field helps overcome the potential barrier. For small voltages, IV characteristics in the barrier limited regime can be linear, but for the large range (0.1 V- 1.0 V) we investigate here, it should be superlinear. For small strain, translocation barriers are the dominant factors and thus we see the superlinear behavior. As we increase the strain, the translocation through the pore becomes barrierless for $q_O = -0.54\,e$. Thus, the ionic current becomes sublinear (diffusion-limited). The approximately Ohmic behavior we observe for 1 % strain looks to be a fortuitous cancellation of the two effects.

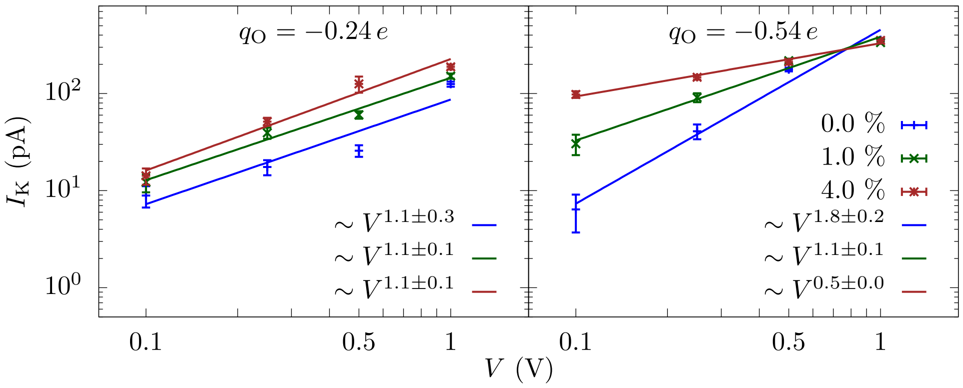

Figure S1. IV-characteristics. K^+ current through graphene crown ether pore at different strains in 1 mol/L KCl versus voltage. When current is limited by barriers, the IV-characteristics are super-linear and, when current is limited by diffusion, the IV-characteristics are sub-linear. Linear behavior may appear due to the cancellation of the two effects. While some of the data fit well to a simple power law, there are prominent features due to the rather complex dependence of energetics on voltage. The error bars are plus/minus one SE from five parallel runs.

Golden aspect ratio: The role of the bulk in determining resistance—specifically in access or diffusion limitations—requires a careful treatment of the simulation cell, as the bulk only slowly converges. We thus employ the golden aspect ratio method [2,3]. This employs a special aspect ratio that, after the disappearance of non-scaling finite-size effects, converges immediately to the infinite bulk limit. Figure S2 shows the current versus the simulation cell cross-sectional length (with height proportional to this length according to the golden aspect ratio). While there is some variation in the current over the length scales shown, it is mostly within the statistical error bars (from five parallel runs). Some of this variation may be non-scaling finite size effects, but overall the change is within expected errors.

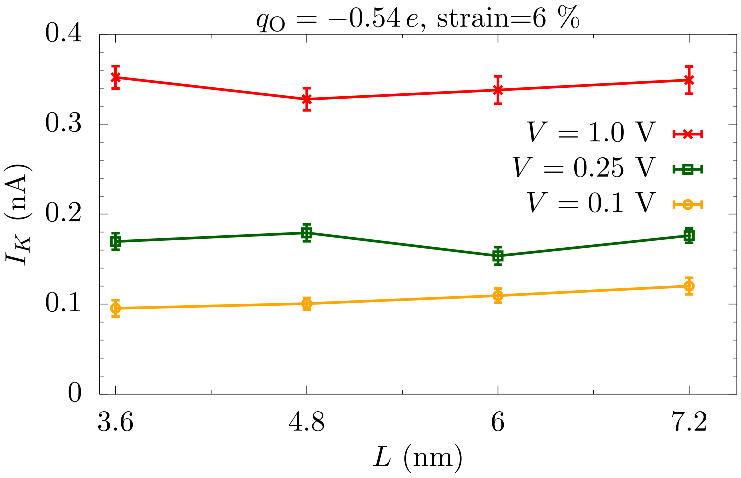

Figure S2. Potassium current through the crown ether graphene pore with various simulation cell cross-sectional lengths but with the same aspect ratio ($H/L \approx 1.2$) at $q_O = -0.54\,e$ and strain 6 %, showing that the ionic current does not vary with simulation cell size as long as the aspect ratio is kept at the golden aspect ratio. The error bars are plus/minus one SE from five parallel runs.

Concentration effects: In the main text, we discuss how for $q_O = -0.54\,e$ at small strain and low voltage the current displays many-body blockade effects. Otherwise, the other parameter regimes are single-ion transport. One way to see many-body effects is to study the concentration dependence of the current. Figure S3 shows the normalized current versus the concentration. For $q_O = -0.54\,e$ at small strain, this normalized current decreases versus concentration, which suggests that many-body effects are at play (this dependence is just outside the error bars). Increasing the concentration does not increase the current proportionally since the main pore site is already occupied and is preventing further current flow. All other cases show only small variations or small upward trend, albeit barely out of the range of the statistical error bars from the five parallel runs.

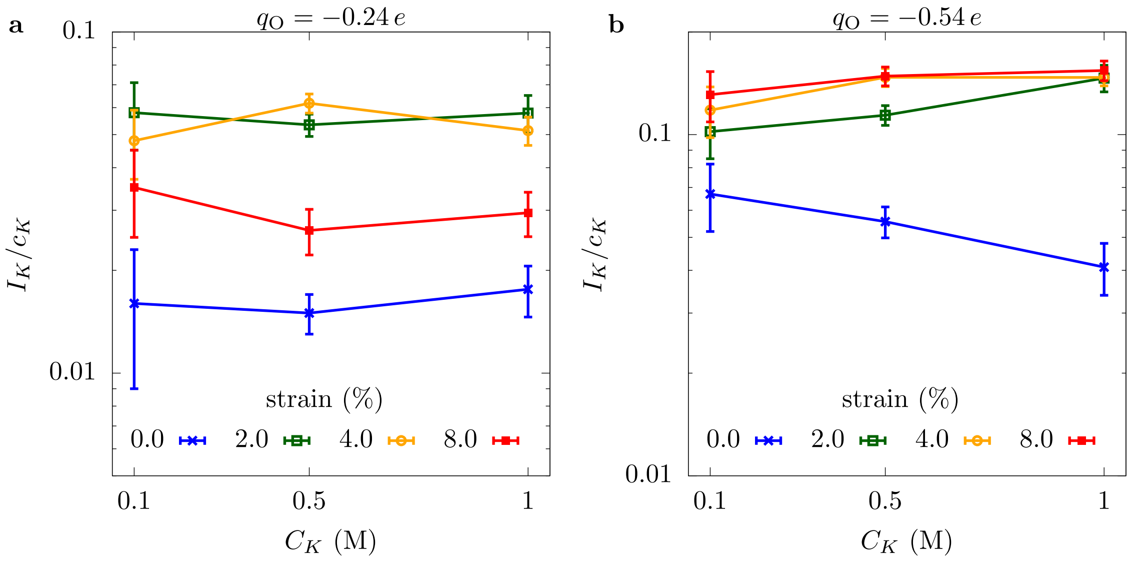

Figure S3. Potassium current normalized by the bulk ion concentration versus the concentration. I_K/C_K remains constant for all the cases where the ionic current is limited by association rate. Only for the unstrained pore with $q_O = -0.54\,e$, where ionic current is influenced by the dissociation rate, does the normalized current decrease with concentration. The error bars are plus/minus one SE from five parallel runs.

Occupancy: Within the main text, we develop a three site model for the current through the $q_O = -0.54\,e$ pore. The form of this model is motivated by concentration and rate data. However, it still has several parameters. To reduce the number of parameters, we compute the occupancy of the main pore site versus strain and voltage. As an input, this allows a more rigorous treatment of the fitting of the MD current data to the model. Figure S4 shows the potassium occupancy of the main pore site versus strain for the four voltages we study. The occupancy in the $q_O = -0.24\,e$ pore does not (except at low voltage) display any well-defined trends. At low voltage and within the main pore region, the free energy is increasing (the satellite barriers are decreasing), giving rise to an exponential decrease in pore occupancy. For other voltages, there is a more complex interplay of strain and voltage within the free energy landscape. However, for $q_O = -0.54\,e$, there is a clear exponential decrease in the pore occupancy with strain due to the raising of the bottom of the potential well. Moreover, the occupancy decreases a bit faster than exponential with voltage (in the main text, we model the dissociation time as Ve^{vV}, with v some positive constant, which is both physically motivated—the exponential represents a decrease in barrier height and the pre-exponential the local field driving the ions—and works well).

Here, we also show additional concentration data versus spatial position, see Figure S5 and Figure S6. This shows where the concentration can increase or decrease with voltage depending on the pore characteristics. It also motivates the three site model we take in the main text.

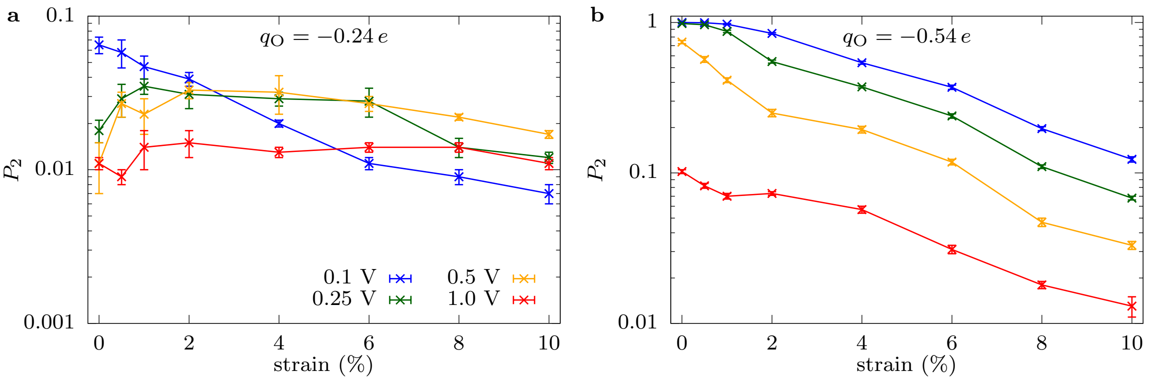

Figure S4. Average number of K^+ ion in the pore ($|z| \leq 0.2$ nm) versus strain for **a.** $q_O = -0.24\,e$ and **b.** $q_O = -0.54\,e$ and for various applied bias. Note that this data includes 0.5 % and 1 % strain, where the main text only includes data at 2 % increments.

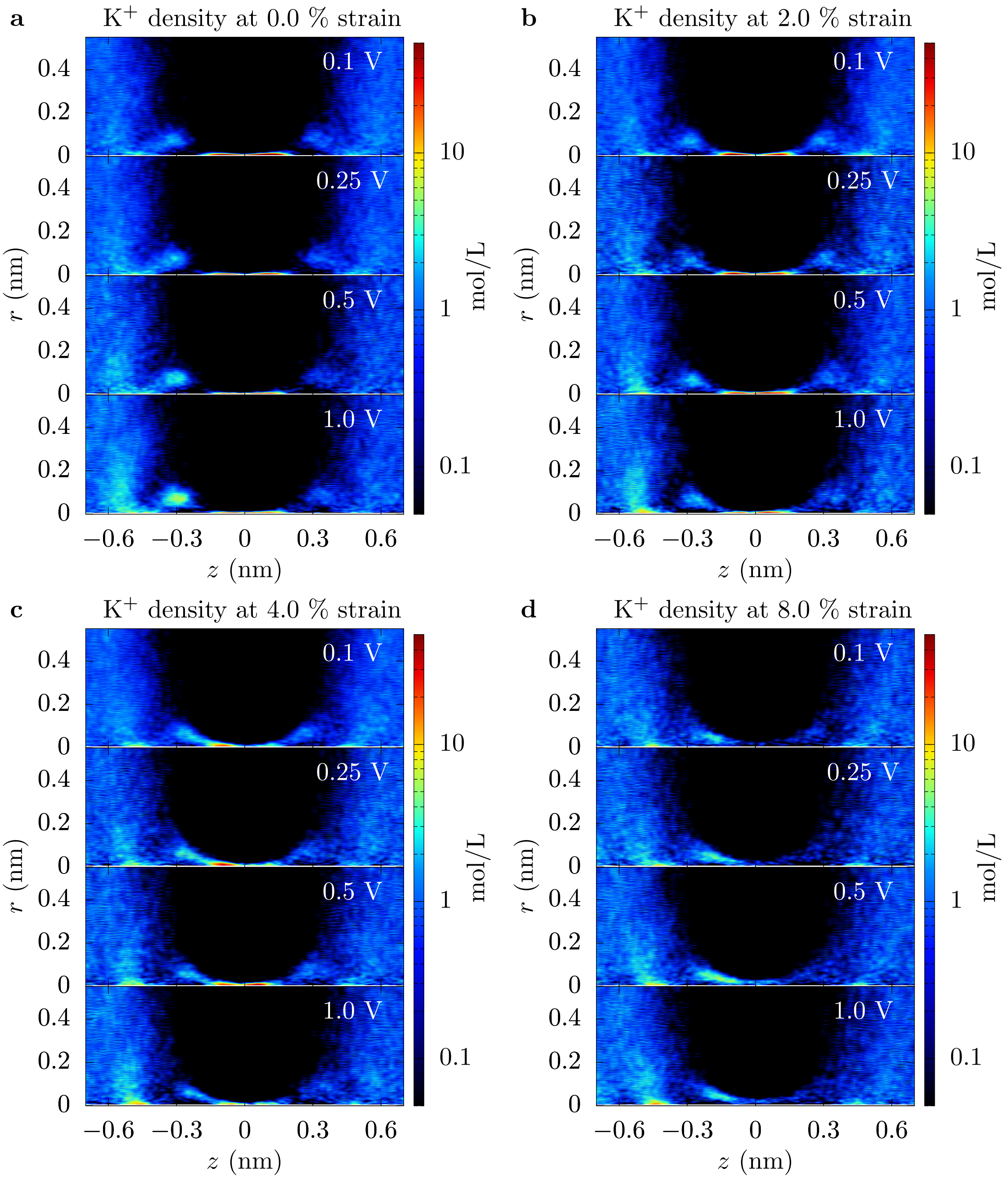

Figure S5. Concentration of potassium ions near a graphene crown ether pore with $q_O = -0.24\,e$ at **a** 0 %, **b** 2 %, **c** 4 %, and **d** 8 % strain for various voltages.

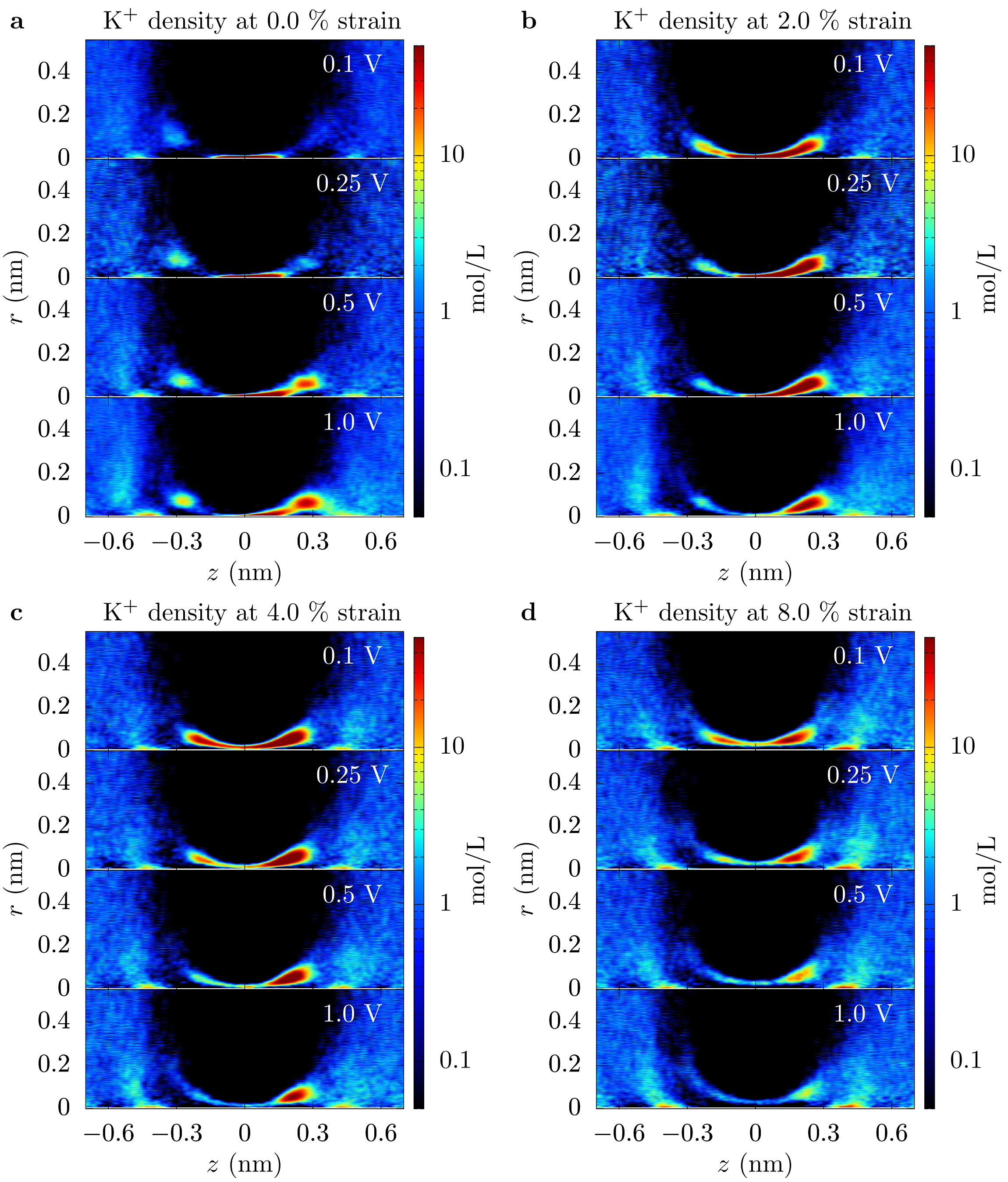

Figure S6. Concentration of potassium ions near a graphene crown ether pore with $q_O = -0.54\,e$ at **a** 0 %, **b** 2 %, **c** 4 %, and **d** 8 % strain for various voltages.

Free energies and electrostatic potentials: Figures S7 and S8 show additional free energy profiles, as well as a larger range of z. These demonstrate that there are indeed irrelevant features in free energy. Specifically, $q_{\mathrm{O}} = -0.54\,e$ has a feature at 0.2 nm for most strains that changes little when the voltage is taken from 0 V to 0.25 V. Moreover, the free energy is near barrierless for the largest strain examined. For $q_{\mathrm{O}} = -0.24\,e$, the barrier in the middle of the pore increases with strain until somewhere between 4 % and 6 % strain, after which the peak mostly broadens and then decreases. This is due to the decrease of the electrostatic interaction initially being unable to compensate for dehydration. At 0.25 V, the pore is nearly barrierless at high strain, as the bias effectively wipes out the main feature.

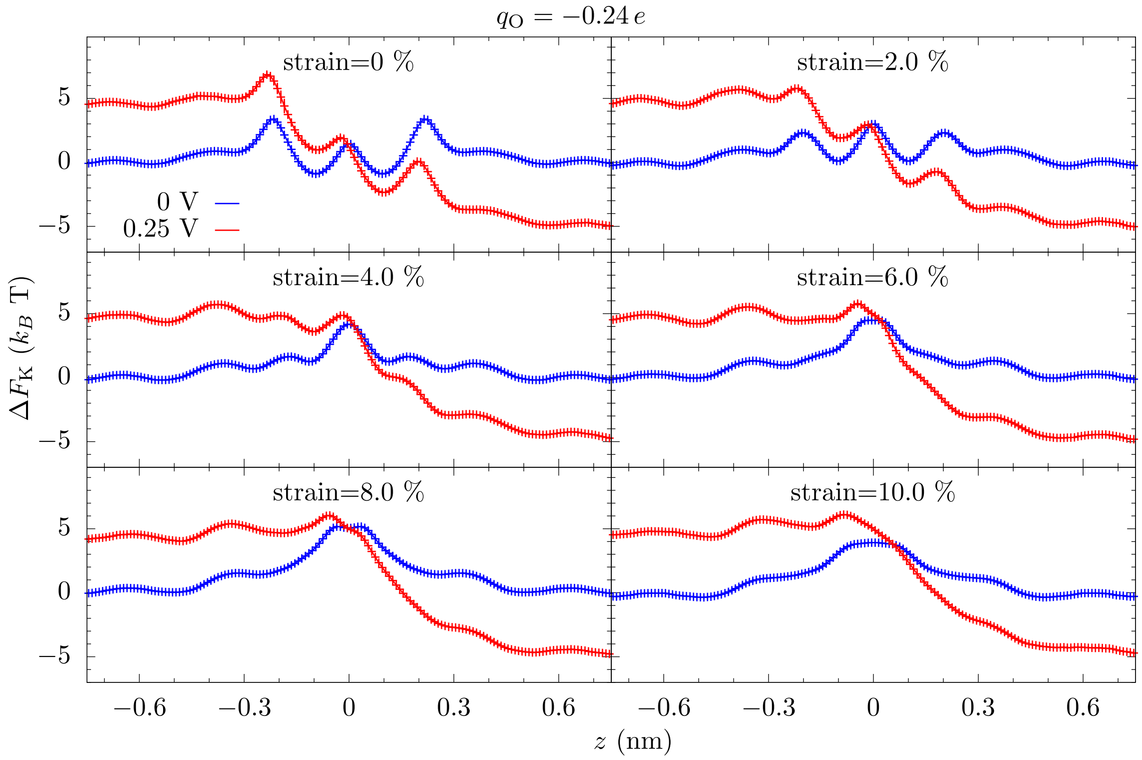

Figure S7. The free-energy profile of K^+ going through a graphene crown ether pore with $q_0 = -0.24\,e$ at various strains for equilibrium and non-equilibrium ($V_{\mathrm{ext}} = 0.25$ V) cases. The error bars are plus/minus one SE from five parallel runs.

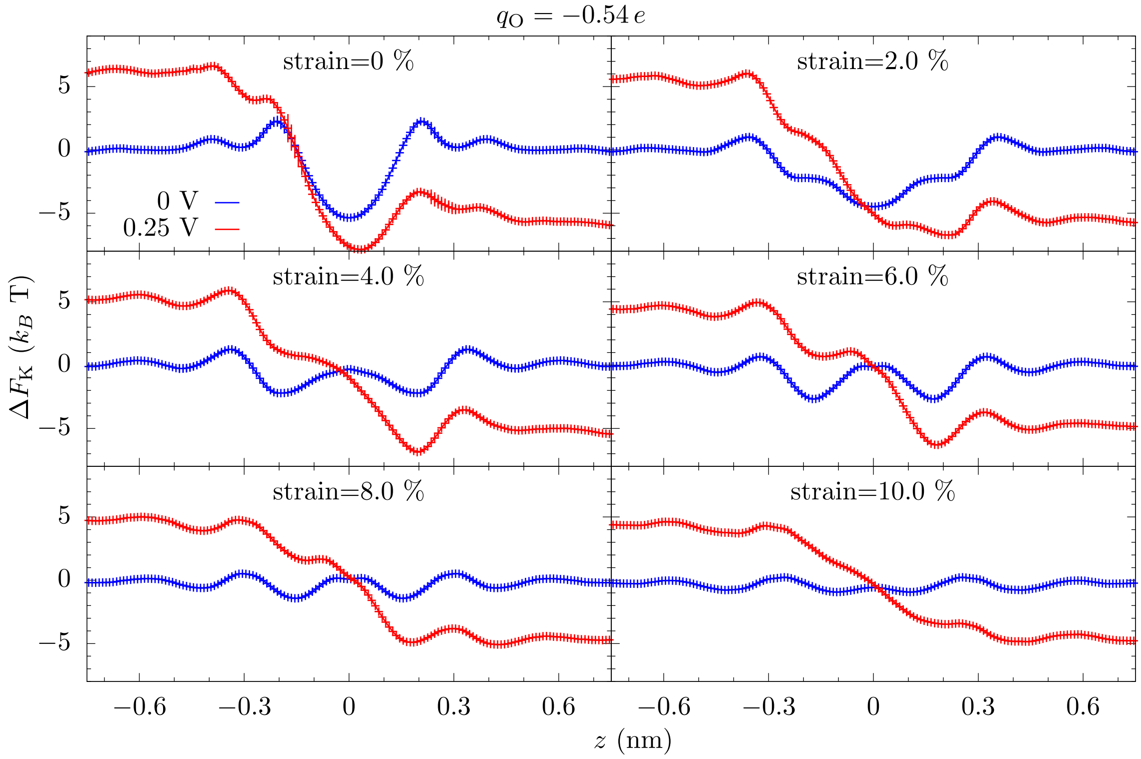

Figure S8. The free-energy profile of K^+ going through a graphene crown ether pore with $q_0 = -0.54\,e$ at various strains for equilibrium and non-equilibrium ($V_{ext} = 0.25$ V) cases. These plots show that the $q_0 = -0.54\,e$ pore veers toward a barrierless configuration, with 10 % strain removing barriers nearly completely. Moreover, it also demonstrates that at 0.25 V, most of the cases (all except 0 % strain) have irrelevant features in the free energy landscape, ones that remain unchanged when voltage is brought to 0.25 V from 0 V. The error bars are plus/minus one SE from five parallel runs.

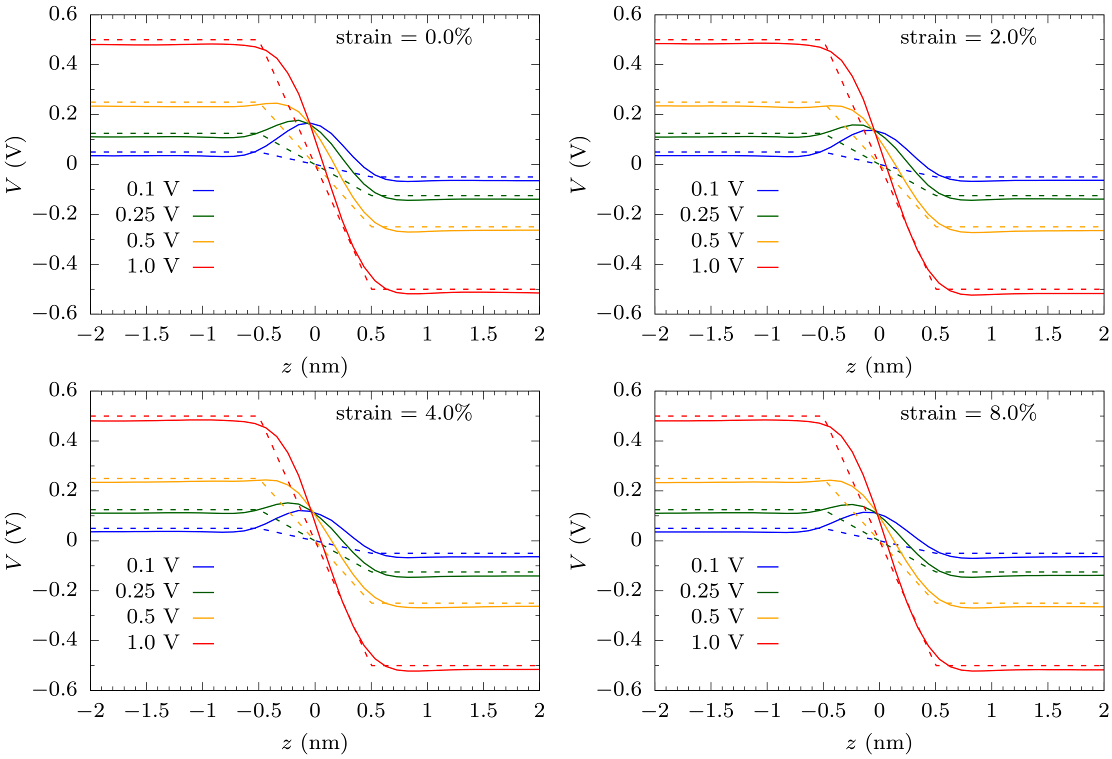

Figure S9. Potential drop along the z−axis in the pores with $q_O = -0.24\,e$ and various strains for different applied voltages. The dashed line shows the model where V drops uniformly between $|z| \leq 0.5$ nm and constant outside it.

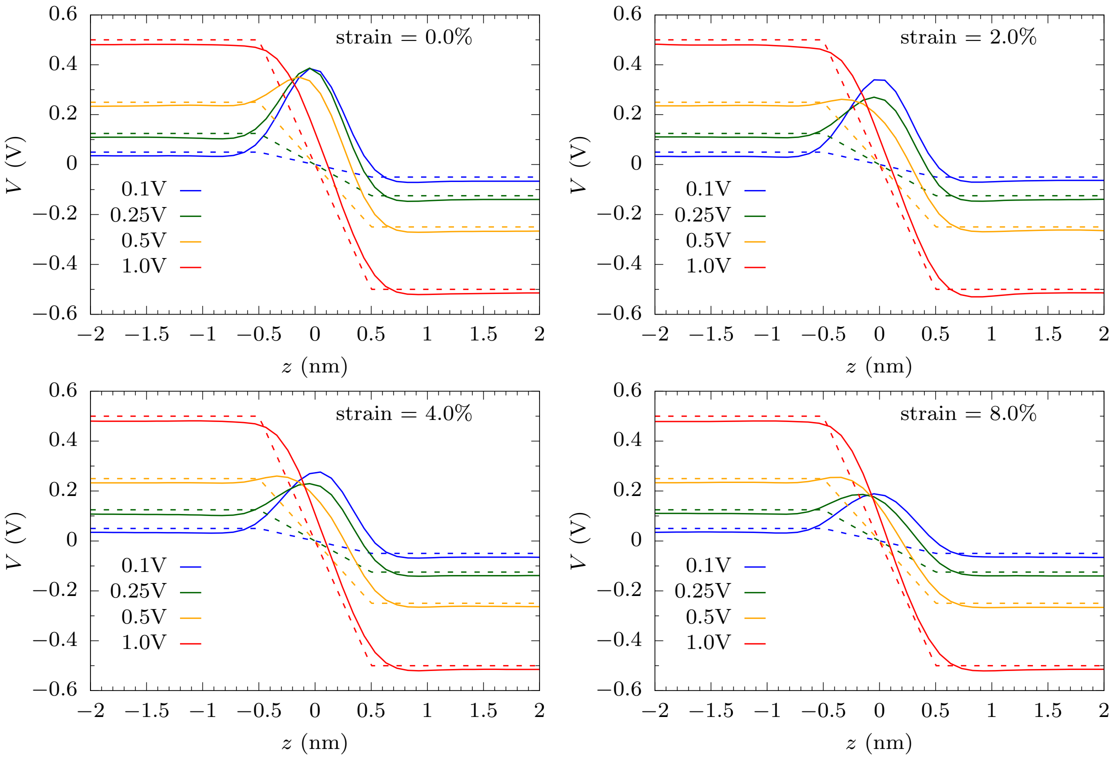

Figure S10. Potential drop along the z−axis in the pores with $q_O = -0.54\,e$ and various strains for different applied voltages. The dashed line shows the model where V drops uniformly between $|z| \leq 0.5$ nm and constant outside it.

Other: In the remaining figures of the SM, we show additional one-way rate data. Figures S11 and S12 show the one-way rate data as in the main text (across *z*-planes) for the rest of the parameter regimes. Figure S13 instead shows one-way rate data across hemispherical surfaces.

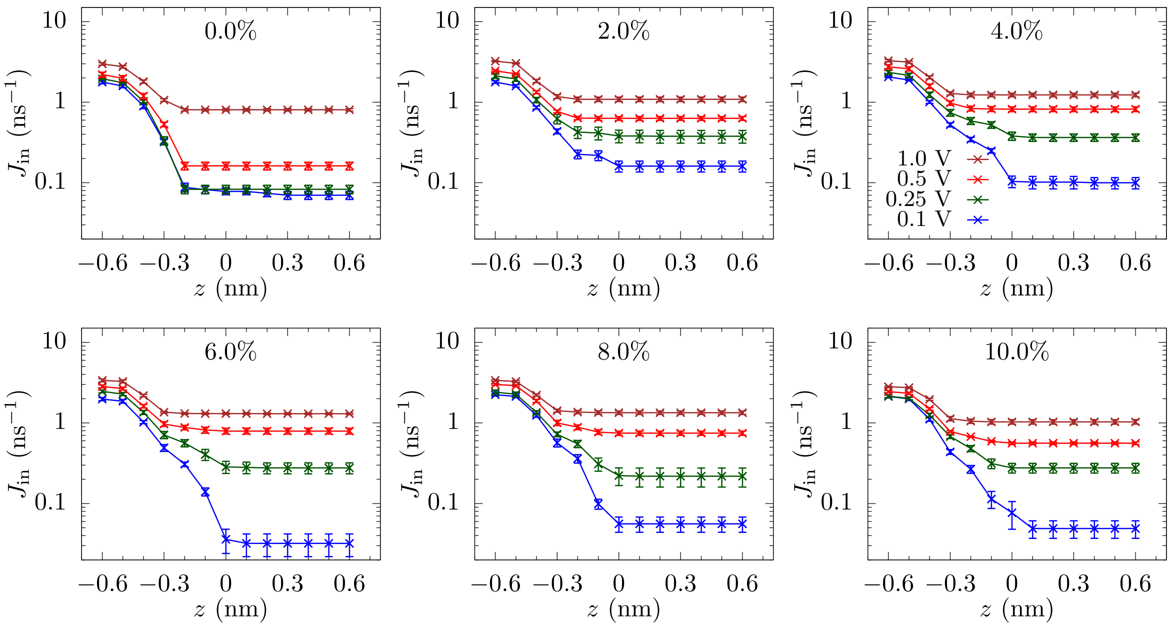

Figure S11. The inward flux of K^+ ions versus z-distance at different applied voltage for pore with $q_O = -0.24\,e$. The error bars are plus/minus one SE from five parallel runs.

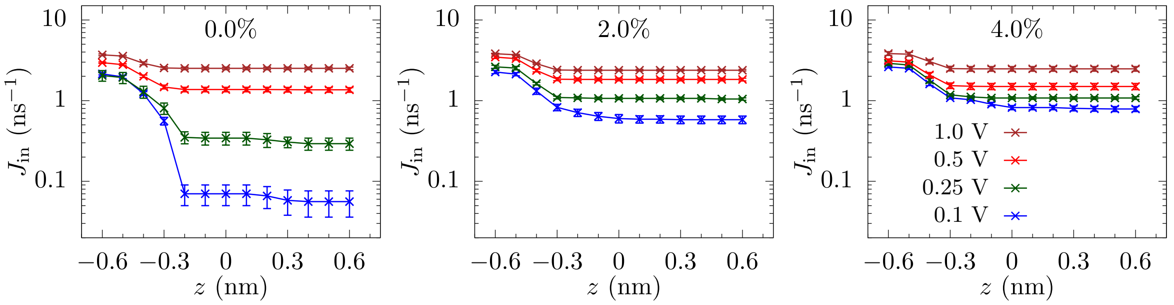

Figure S12. The inward flux of K^+ ions versus z-distance at different applied voltage for pore with $q_O = -0.54\,e$. The error bars are plus/minus one SE from five parallel runs.

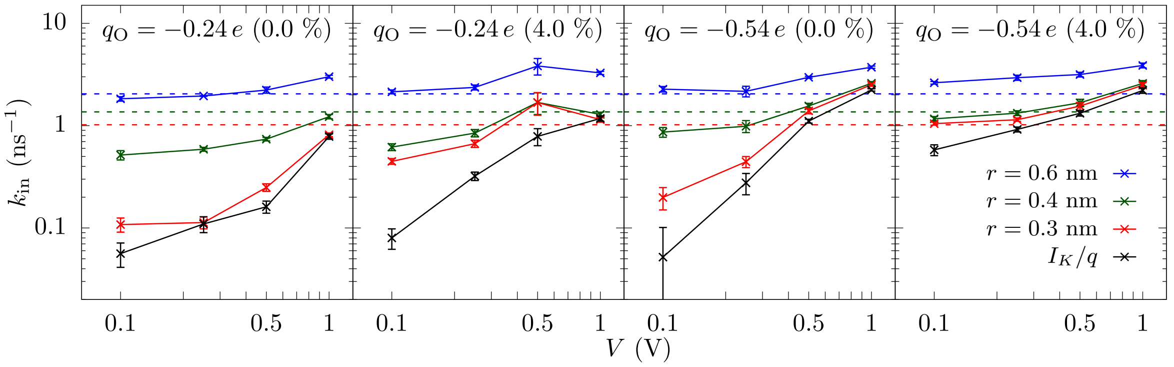

Figure S13. Diffusion and barrier limited currents. The inward diffusion rate of K^+ at a distance 0.6 nm, 0.4 nm, and 0.3 nm from the pore versus applied voltage. The dashed horizontal lines give the rate from the diffusion equation assuming ions only enter through a quarter of the sphere (i.e., $\pi\, D\, c\, r$). For small voltage, the $k_{\rm in}$ near the pore is much smaller than diffusion limit and thus the current is limited by the barrier to transport. At large voltage, $k_{\rm in}$, and hence the current, approach the diffusion limit. The error bars are plus/minus one SE from five parallel runs.

1. Nelson, P.H. A permeation theory for single-file ion channels: One-and two-step models. *J. Chem. Phys.* **2011**, *134*, 04B615.
2. Sahu, S.; Zwolak, M. Maxwell-Hall access resistance in graphene nanopores. *Phys. Chem. Chem. Phys.* **2018**, *20*, 4646 – 4651.
3. Sahu, S.; Zwolak, M. Golden aspect ratio for ion transport simulation in nanopores. *Phys. Rev. E* **2018**, *98*, 012404.

© 2020 by the authors. Licensee MDPI, Basel, Switzerland. This article is an open access article distributed under the terms and conditions of the Creative Commons Attribution (CC BY) license (http://creativecommons.org/licenses/by/4.0/).
